# Feeling the heat: Elevated temperature affects male display activity of a lekking grassland bird

**DOI:** 10.1371/journal.pone.0221999

**Published:** 2019-09-16

**Authors:** Mishal Gudka, Carlos David Santos, Paul M. Dolman, José Mª Abad-Gómez, João Paulo Silva

**Affiliations:** 1 School of Biological Sciences, University of East Anglia, Norwich, United Kingdom; 2 Núcleo de Teoria e Pesquisa do Comportamento, Universidade Federal do Pará, Belém, Brazil; 3 Department of Migration, Max Planck Institute for Animal Behavior, Radolfzell, Germany; 4 School of Environmental Sciences, University of East Anglia, Norwich, United Kingdom; 5 Conservation Biology Research Group, Department of Anatomy, Cell Biology and Zoology, Faculty of Sciences, University of Extremadura, Badajoz, Spain; 6 Servicio de Conservación de la Naturaleza y Áreas Protegidas, Consejería de Medio Ambiente y Rural, Políticas Agrarias y Territorio, Junta de Extremadura, Mérida, Badajoz, Spain; 7 CIBIO/InBio, Centro de Investigação em Biodiversidade e Recursos Genéticos, Laboratório Associado, Universidade do Porto, Campus Agrário de Vairão, Vairão, Portugal; 8 CIBIO/InBio, Centro de Investigação em Biodiversidade e Recursos Genéticos, Laboratório Associado, Instituto Superior de Agronomia, Universidade de Lisboa, Tapada da Ajuda, Lisbon, Portugal; University of Sassari, ITALY

## Abstract

Most species-climate models relate range margins to long-term mean climate but lack mechanistic understanding of the ecological or demographic processes underlying the climate response. We examined the case of a climatically limited edge-of-range population of a medium-sized grassland bird, for which climate responses may involve a behavioural trade-off between temperature stress and reproduction. We hypothesised that temperature will be a limiting factor for the conspicuous, male snort-call display behaviour, and high temperatures would reduce the display activity of male birds.

Using remote tracking technology with tri-axial accelerometers we classified and studied the display behaviour of 17 free-ranging male little bustards, *Tetrax tetrax*, at 5 sites in the Iberian Peninsula. Display behaviour was related to temperature using two classes of Generalized Additive Mixed Models (GAMMs) at different temporal resolutions. GAMMs showed that temperature, time of the day and Julian date explained variation in display behaviour within the day, with birds snort-calling significantly less during higher temperatures. We also showed that variation in daily snort-call activity was related to average daytime temperatures, with our model predicting an average decrease in daytime snort-call display activity of up to 10.4% for the temperature increases projected by 2100 in this region due to global warming. For lekking birds and mammals undertaking energetically-costly displays in a warming climate, reduced display behaviour could impact inter- and intra-sex mating behaviour interactions through sexual selection and mate choice mechanisms, with possible consequences on mating and reproductive success. The study provides a reproducible example for how accelerometer data can be used to answer research questions with important conservation inferences related to the impacts of climate change on a range of taxonomic groups.

## Introduction

Climate change is a major and increasing global threat to biodiversity [1,2], therefore a better understanding of species responses to climate change is important to inform and prepare policy makers and conservationists [3–5]. However, most climate-species models relate range margins to long-term mean climate variables on assumed equilibrium, and do not adequately capture responses to increasing climatic extremes and stochasticity [6,7]. More fundamentally, such models lack a mechanistic understanding of the ecological, behavioural and demographic processes that currently limit range distributions and how these processes may respond to future climates. This limits their ability to predict future range suitability and responses [8]. Here we examine how global warming responses may involve a behavioural trade-off between avoiding temperature stress and carrying out breeding displays, for a climatically limited, edge-of-range population of a medium-sized grassland bird.

For some ectotherms such as reptiles and amphibians, the impacts of elevated temperatures on species’ behaviour patterns are well evidenced, and local population expiration can occur when temperature increases restrict key activities to a limited daily interlude [3]. In contrast for endotherms, understanding of how physiological and behavioural mechanisms contribute to climate range limits is largely lacking. For endotherms, higher temperatures can affect individuals physiologically, leading to higher metabolic rates and possible overheating [9]. Indeed, mass deaths of bats and birds related to heat waves have been reported [10]. A common response to mitigate impacts of temperature is behavioural thermoregulation [11], where animals remain mostly inactive in sheltered locations [12]. This response has been found, for example, in subterranean rodents which reduce above-ground activity [13] and baboons (*Papio hamadryasursinus*) which reduce feeding activity and increase grooming and resting behaviour [14]. This reduced activity, if prolonged, may impact individual fitness and thus wider population dynamics [15]. Understanding the potential impacts of climate warming on endotherms will help biologists to identify the most threatened species, better forecast their future distributions [16–19], better understand the mechanistic stressors, and subsequently respond more effectively to conserve them [4]. Species restricted to open grassland, semi-arid and desert habitats may be particularly vulnerable to global warming, as they are highly exposed to insolation, with limited ability to perform behavioural thermoregulation due to the open nature of the landscape that they depend on.

In species showing extravagant sexual display, such as lekking breeding systems, where one sex (usually the males) display in clustered territories that are visited by the opposite sex for the sole purpose of breeding [20], reduced breeding activity caused by high temperatures may have both inter- and intra-sex implications [21,22]. In many lekking ungulates (including Red deer *Cervus elaphus*, Fallow deer *Dama dama*, Blackbuck *Antilope cervicapra* and Uganda kob *Kobus kob*), males undertake energetically costly display behaviours, such as powerful vocalisation (bellowing, snorting), displays of physical strength and fighting, that increase their chance of obtaining copulations [23–26]. In African houbara, *Chlamydotis undulata*, male display activity reliably indicates health status and ejaculation quantity and quality of males, allowing females to optimize fertilization success and the genetic quality of offspring [27–29]. Female houbara were also shown to adjust maternal allocation according to the observed male display rate, with consequences for hatching and fertilization success and chick growth and survival [21].

The little bustard, *Tetrax tetrax*, is a lekking grassland bird that has been shown to reduce its overall activity at high temperatures in SW Iberia [30]. This area has some of the highest temperatures in Europe, and is also expected to be particularly affected by global warming and severe drought episodes by the end of the twenty-first century [31]. Males use energetically-costly ‘snort-calling’ to signal their presence (and potentially their quality) to females [22], and as an intra-sexual display addressed to competing breeding males [27,32].

The use of modern, affordable technology such as GPS loggers and accelerometers, is now allowing for novel methods to remotely collect accurate, high-resolution animal movement and positional data to provide increased insights into species behaviours and climate-related movement [33]. We investigated how elevated temperatures affected snort-calling of little bustard males, by remotely tracking seventeen free-ranging male birds during the mating season and classifying individual behaviour from fine-scale movement data collected using tri-axial accelerometers. The frequency of ‘snort-call’ display activity classified from these acceleration sequences were related to ambient temperature. We hypothesised that temperature would be a limiting factor for display behaviour, and that high temperatures would reduce the display activity of male birds.

## Materials and methods

### Study species and study site

The global breeding range of the little bustard (IUCN Near-Threatened) is confined to the Palearctic region including west and southern Europe, central Asia and north Africa [34]. Originally a steppe bird, it is presently well-adapted to extensive agriculture and pastures [35]. Its climatic niche in southern Europe seems to be more restricted than other bustard species, such as the great bustard (*Otis tarda*), avoiding areas with greater temperature extremes [36]. Within Europe the species is classified as ‘Vulnerable’ because of recent sharp population declines [34] mainly due to agricultural intensification and loss of preferred extensively-managed, heterogeneous farmland [37,38]. The little bustard’s western European stronghold is found in the Iberian Peninsula [39].

**Fig 1:**
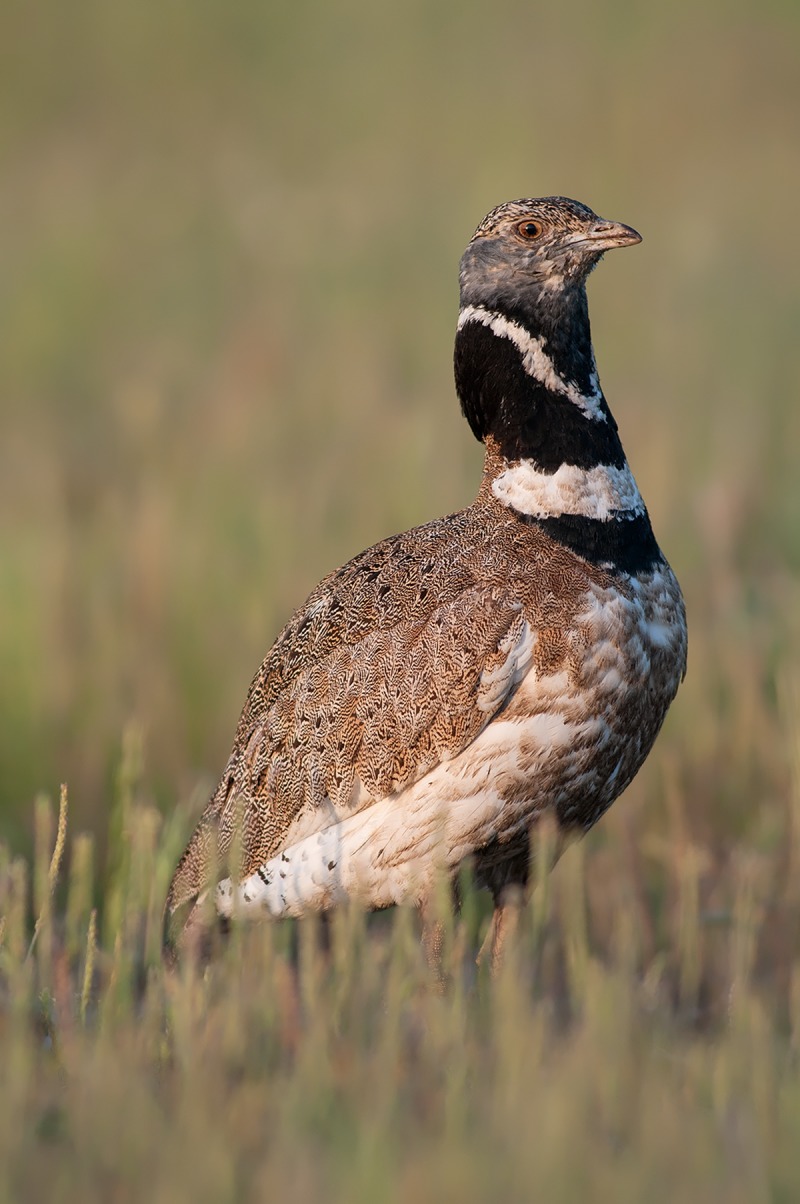
Male little bustard performing its characteristic snort-call display.

Our study was conducted in two regions of south-western Iberia: Alentejo (Portugal) and Extremadura (Spain) that have a thermo- and meso-Mediterranean climate [40] with warm, dry summers and cold, humid winters [41]. The average daily daytime (5:00–21:00 hours) temperatures in our study region varied between 10 ºC and 31ºC during our study period of April and May 2014 and 2015. The little bustard’s mating season in south-western Iberia usually starts in the beginning of April and extends until mid/late May [42], with female brood-rearing continuing until the end of June. Males have a conspicuous and likely energetically costly display ritual that is used to defend their territories or ‘display centres’ from other opportunistic males [43] and make them more visible to females. It is mainly characterised by standing relatively upright, puffing up their necks, and uttering a snort-call (Fig 1) and is by far the most common behaviour related to breeding display [27,44].

### Data collection

**Fig 2:**
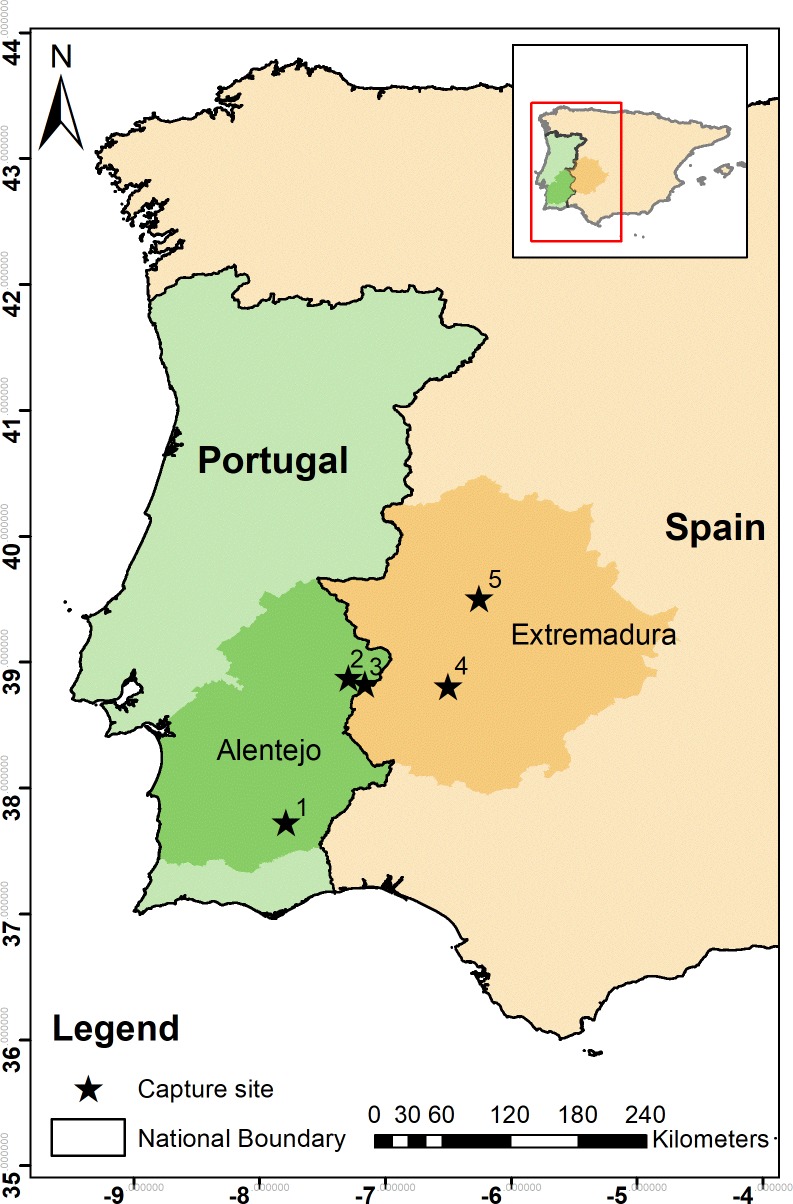
**Location of the five study sites where 17 male little bustards were captured, tagged and tracked in the regions of Alentejo and Extremadura (darker shade) in Portugal (green) and Spain (orange) respectively, during the display seasons (April and May) of 2014 and 2015.** 1- SPA Castro Verde; 2 –SPA Vila Fernando; 3 –SPA Torre da Bolsa; 4 –Arroyo de San Servan; 5 –SPA Llanos de Cáceres (SPA–Special Protection Area).

In April and May 2014 and 2015, 17 wild adult breeding males (13 birds at 3 sites in Alentejo, Eastern Portugal and 4 birds at 2 sites in Extremadura, Western Spain. Fig 2) were captured using snares at their breeding grounds and fitted with solar-powered GPS-GSM-accelerometer tracking devices (Movetech Telemetry, UK, www.movetech-telemetry.com). To reduce the risk of increased energy expenditure and alterations to movement patterns, the loggers weighed less than 3.2% of the mass of the bird (approximately 24 g) [45–48] and were aerodynamically designed without a protruding antenna to minimise drag [49]. Birds were tagged using a full (‘back-pack’) harness made from 6mm wide Teflon ribbon, with adjustable straps so that loggers could be positioned between the wings on the back, at the centre of mass of the bird, to minimise effects on flight patterns [50]. The harness was stitched at one point under the sternum and designed to release within a year. The device and straps were coloured to blend with the brown feathers on the bird’s back. Bird trapping and GPS tagging were approved by the Instituto da Conservação da Natureza e Florestas (Portuguese authority) through licenses to João Paulo Silva (ICNF/CAPT/2014, ICNF/CAPT/2015) and Consejería de Medio Ambiente y Rural, Políticas Agrarias y Territorio of the Junta de Extremadura (Spanish authority) through the license to José Mª Abad-Gómez.

The onset of the display and hence capturing season was determined from field observations, noting when males first started exhibiting full display behaviour [27,42]. Capturing was generally attempted during the two periods of greatest bird activity; just after dawn and a few hours before dusk. Tracking devices were fitted in less than 20 minutes to reduce the chances of the birds suffering from potentially fatal capture myopathy [51].

Tracking devices contain a tri-axial accelerometer which measures acceleration along the sway, surge and heave axes in ten-second data bursts of 1 Hz (collected every 10 or 20 minutes depending on program settings) and remotely transmit these data (hereafter ‘acceleration sequences’) via GSM (Global System for Mobile communication). Accelerometers could read up to ±6G, which proved more than enough to characterize behaviours, as readings only rarely got close to 3G during flight events. Ambient temperature was measured every hour, using a temperature datalogger placed near the ground at each successful capture site. For 2 birds (out of 17) we obtained data from meteorological stations located within 10 km of the capturing site, as temperature loggers were not successfully retrieved from these sites.

### Classifying behaviour from accelerometer data

Thirteen accelerometer sequences were validated in May 2015 using concurrent video footage of two tagged birds snort-calling in their natural circumstances [52,53]. Validating accelerometer sequences directly from wild animals is considered the most accurate method for calibration [54], and is relatively uncommon, as many studies instead use captive animals or surrogate species [55]. The 13 accelerometer sequences were characterised by mean (± sd) heave values of 0.60g ± 0.10, mean sway values of -0.03g ± 0.03, and mean surge values of 0.81g ± 0.09 (S2 Fig). Although similar accelerometer readings e.g. high values of heave, may also be observed when the bird becomes alert and upright due to disturbance, we assumed that during the mating season, the time spent alert due to disturbance would be minimal compared with the time spent in display. This is supported by field observations of the birds across several display seasons (Silva, pers observation) and because breeding behaviour is density-dependent [22], the rate of snort call is expected to be greater at our high-density sites compared to the 3.8 calls per minute observed in lower-density sites in France [27].

Using these predetermined criteria, 1356 randomly selected accelerometer sequences (10% of the total) were manually classified as either display behaviour or not, based on the predominant behaviour within the first 7 seconds. Using the software ‘AcceleRater’, these training data were used to compute a range of summary statistics (Mean, Standard deviation, Skewness, Kurtosis, max, min, the vector norm of the measurement, covariance between two axis, Pearson correlation between two axis, dynamic body acceleration, the average difference between two axis, 25%, 50%, 75% percentiles etc.) to build multiple classification models [56,57]. ‘AcceleRater’ runs seven types of classification models with different characteristics: Artificial Neural Network; Decision Tree; Linear Support Vector Machine; Linear/Quadratic Discriminant Analysis; Nearest Neighbours; Radial Basis Function kernel Support Vector Machine and Random Forest. Performance of the resulting models were assessed by their ability to classify an independent validation training set using a k-fold cross-validation method applied across the set of 1356 manually classified sequences. The training dataset was randomly split into k equal-size parts, k-1 parts are used for training and 1 for validation. The procedure was repeated k-times until all parts have been used for validation. We found that a value of 10 for k produced the highest accuracy. The best performing model, the Random Forest model, was then used to automatically classify the remaining 90% of data (S1 Fig).

### Modelling snort-call display behaviour

Accelerometer data were analysed for the period commencing two days after the loggers were fitted on the bird (to ensure normal behaviour was resumed) and ending when males left their territories for the first migratory movements (using GPS data collected by the dataloggers). Data collected between 21:00 hours and 5:00 hours were excluded from analyses, as birds display minimally at night [30]. Display behaviour was related to temperature in two classes of model at different temporal resolutions, (1) within days, using hourly measurements and (2) between days, using daily averages (see S1 Table for variable details). We used Generalized Additive Mixed Models (GAMMs) as we expected complex non-linear responses of display [30] and included random effects to control for non-independence of repeated measures. The first model related snort-call display behaviour occurrence (0/1) for each accelerometer sequence to fixed smooth effects of temperature (mean for the hour behaviour was recorded), time (continuous) and Julian date (a sequential count of the number of days since the start of the year), using binomial error structure, also incorporating the random effect of bird identity. Correlations between predictors were checked prior to analysis to prevent multi-collinearity (temperature vs time: r = 0.51; temperature vs Julian date: r = 0.16; time vs Julian date: r = 0.01). Smooth functions in GAMMs remove the autocorrelation structure from the data, leaving residuals that are independent [58].

For the second model, we omitted any day that a bird did not provide at least two accelerometer readings per hour for a minimum of 11 of the 16 daylight hours (6144 accelerometer sequences remaining). This was to ensure that data were representative and consistent across the day i.e. enough samples from different parts of the day, to provide a representative daily average. From this filtered dataset, for each bird we calculated daily (5:00–21:00 hours) (a) mean daytime temperature using all temperature data obtained at the site and (b) proportion of display sequences (hereafter ‘display activity’), averaged from hourly proportions to reduce any bias on the daily average due to unequal number of accelerometer samples recorded across different times of the day. The GAMM related display activity to the fixed effects of mean daytime temperature and Julian date (103 data points), with normal error distribution, incorporating the random effect of bird identity. Multi-collinearity was low with the correlation between mean daytime temperature and Julian date only 0.3. GAMMs were fitted in R (R, 2013) using the function gamm4 of the package gamm4 [59], with the smoother parameter k set to 4 to avoid overfitting [60,61]. Predictive error of both GAMMs was estimated through a 10-fold cross-validation, with the original data randomly split into 10 equal-size parts, 9 parts used to train a new model and 1 to validate the model predictions. This procedure is repeated ten times in a way that training and testing subsets of each run were complementary [62]. For the first model, predictive error was the average of error rates produced for each sub-model of the cross-validation process, with error rate defined as the percentage of misclassifications produced by binomial models. For the second model, predictive error was the average of Normalized Root Mean Square Error, defined as the root mean square error divided by the range of the model response variable.

## Results

The 17 male little bustards were tracked for a mean of 12.8 days (sd: ± 7.2, range: 2–25 days) during their display season, providing 8308 daylight acceleration sequences (5:00 to 21:00 hours), of which 41.5% were classified as snort-call display behaviour (see methods for the classification details). The best combination of precision (probability that an assigned behaviour is correctly classified) and recall (probability that a sample with a particular behaviour will be correctly classified) for classifying snort-calls was obtained using the Random Forest model with k = 10 cross-validation (0.92 and over 0.97 respectively) (S1 Fig).

**Fig 3:**
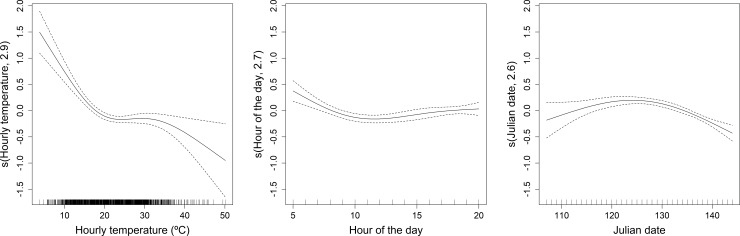
**GAMM partial effects of (left) temperature (centre) time of day (5:00–21:00 hours) and (right) Julian date on the probability of snort-call display.** The GAMM used 8308 7-second accelerometer sequences from 17 male little bustards sampled during the display seasons (April and May) of 2014 and 2015 in SW Iberia. The model incorporates bird identity as a random factor. Dashed lines represent 95% confidence intervals.

The snort-call display probability was significantly related to temperature, Julian date and time of day (Table 1, Fig 3). Snort-call display probability decreased substantially as temperature increased from 4 to 20º C, stabilized from 20 to 30º C, and decreased thereafter (Fig 3). Birds snorted more in the early morning; the display probability declined from 5:00 to 10:00 hours after which it was relatively constant (Fig 3). Display probability increases from day 107 and peaks at day 124, and as the display season continues past this, display probability decreases steadily (Fig 3).

**Table 1:** Summary of GAMMs relating (a) the probability of male little bustard snort-call display behaviour to smoothed fixed effects of hour of the day (time, 5:00–21:00 hours), mean hourly temperature and Julian date; (b) proportion of snort-call display behaviour each day, to smoothed fixed effect of daily average daytime temperature (5:00–21:00 hours) and Julian date. Both models incorporate random effect of bird identity. 17 little bustards were tagged with accelerometers during the display seasons (April and May) of 2014 and 2015 at five sites in Extremadura (Western Spain) and Alentejo (Eastern Portugal). In model (a) predictive error is represented by the average ± standard deviation of the error rates of 10 cross-validation models, while in model (b) we used the average ± standard deviation of the Normalized Root Mean Square Errors. edf denotes estimated degrees of freedom. In both models, display behaviour was classified from seven-second accelerometer sequences.

Parameter	edf	*χ*^*2*^	F	P	Predictive error
(a) Snort-call display probability					37.7 ± 1.5
s(Hourly temperature)	2.9	60.4		<0.001	
s(Hour of the day)	2.7	20.0		<0.001	
s(Julian date)	2.6	51.4		<0.001	
(b) Daily proportion of snort-call display					31.4 ± 5.4
s(Daily mean temperature)	1.6		5.0	0.04	
s(Julian date)	2.0		4.7	0.02	

**Fig 4:**
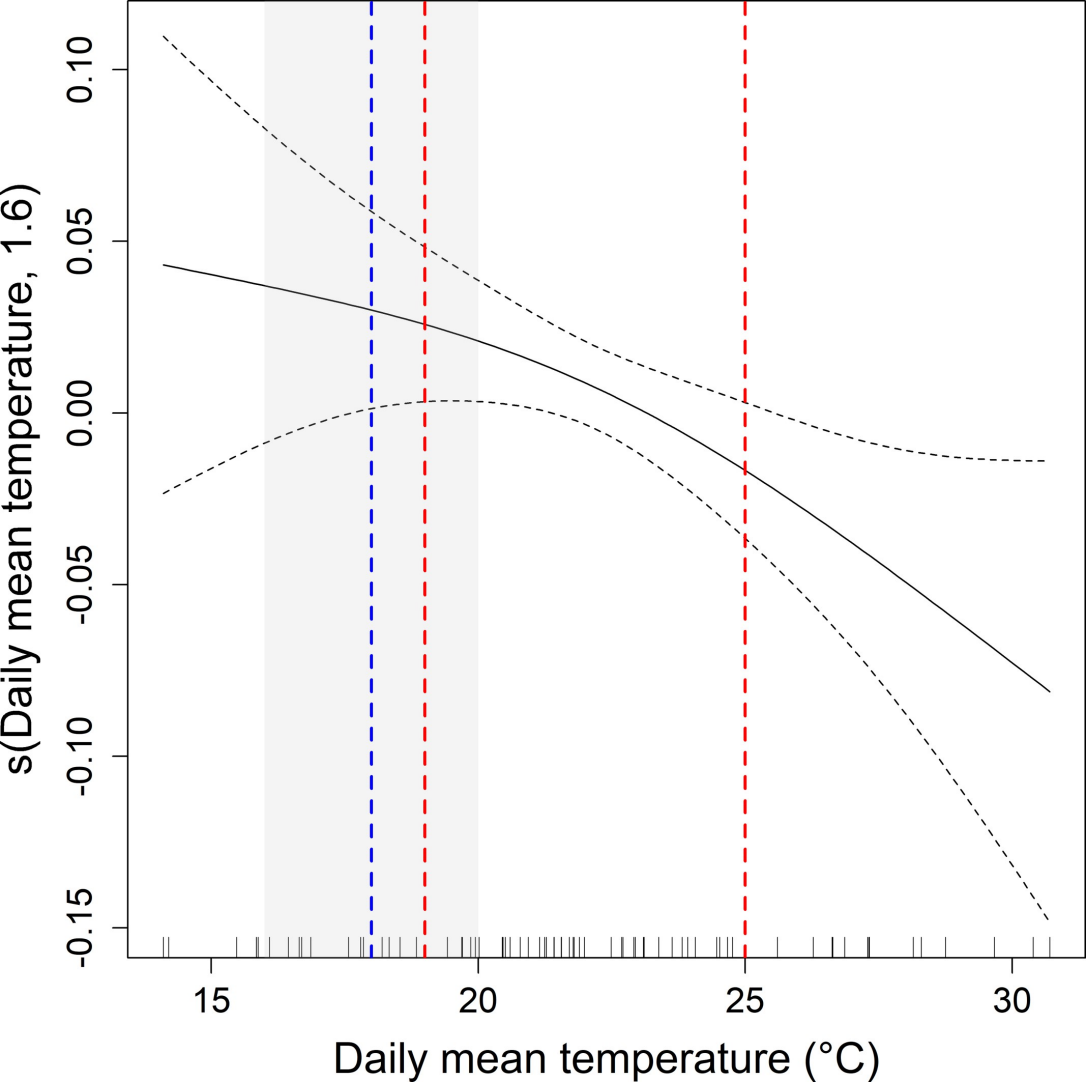
GAMM showing the partial effect of average daytime temperature (5:00–21:00 hours) on display activity (proportion of display events in each day) of 17 male little bustards tagged at five sites in Extremadura (Western Spain) and Alentejo (Eastern Portugal) in SW Iberia. The model incorporates average daytime temperature and Julian date as fixed effects and bird identity as random effect. The blue dashed line indicates the mean daytime temperature across the display season of little bustards in SW Iberia over the last five years (2014–2018) (grey shaded area represents the range of the mean temperatures each year) and red dashed lines indicate the increase in mean daytime temperature based on the 5-year display season average and the best (1°C) and worst (7°C) case IPCC scenarios of temperature increase by the end of the century for Southern Europe/Mediterranean region. Dashed black lines represent 95% confidence intervals.

The daily snort-call display activity (proportion of display events in each day) was related to daily mean daytime temperature and Julian date (Table 1). Display activity decreased with increasing mean daytime temperature (Fig 4) as well as increased to a maximum in the middle of the display season before declining (S3 Fig).

## Discussion

This work provides a reproducible method for predicting how future elevated temperatures may affect a specific aspect of the mating behaviour of a bird species using accelerometer data from advanced remote tracking technology.

The variation of snort-call occurrence of male little bustards during daylight hours was partially explained by temperature, time in the day, and Julian date. Variation in overall levels of daily snort-call activity was also related to average daytime temperatures, which is important given that future scenarios of global warming predict daily mean, rather than hourly, temperatures. Over the last five years (2014–2018), the overall mean daytime temperature across the display season of little bustards in SW Iberia was 18°C (range 16–20.0°C, measurements from a weather station at 38.3284°N, 8.0066°W). Based on this 5-year average and IPCC scenarios which project temperature increases of between 1°C and 7°C (depending on the scenario and the season) by the end of the century for the Southern Europe/Mediterranean region [63], we can assume that average temperatures across the display season may increase to between 19°C and 25°C by 2100. Our model predicts a decrease in average snort-calling activity between 0.9 and 10.4% for temperature changes of this magnitude. Climate change models [31] for the Mediterranean Basin also forecast (with a high level of certainty) a significant increase of heat wave events, which have already pushed maximum temperatures over 40°C during mid-May in recent years. These extreme events may already be curtailing the length of the mating period (Silva, unpublished data) and are therefore likely to compound and exaggerate the decreases in display activity further. Morales *et al*. (2014), also showed that birds have lower snort call rates in poorer habitats, and with the likely decline in habitat quality in the future, we can expect even greater impacts on snort-calls levels.

In mammals and birds, energetically costly displays, calls and other male-male breeding interactions like fights, clashes and chases, play important roles in hierarchy establishment and female monopolization [64,65]. Reductions in such behaviours could potentially lead to less defined hierarchies, thereby allowing floaters (lower ranking, untested sub-adults or less-fit adult males that do not hold territories) more frequent access to females. This could lead to reduced mating success as floaters tend to harass and chase females [22]. In addition, other important display behaviours for male little bustard such as wing flashing and jumping, which are performed more by territorial males [64], are also likely to decrease as they are correlated with snort-calling. These factors, combined with the reduced active daily time of the lek, could result in females being less able to select territorial males with preferred genetic traits, with likely implications on female reproductive success and offspring health and hence population viability of this species [21]. However, the opposite is also possible, if higher temperatures filter out weaker males, allowing fitter and more resilient males with more resistance to the heat greater mating opportunities.

For little bustards, these potential consequences of reduced display combined with other existing threats and additional future climate change related impacts e.g. reduced fitness due to greater thermoregulatory-related energetic losses, may push this endangered species towards local and regional extirpation. Additionally, the link between display effort and mating success has been demonstrated in several lekking birds and mammals, including great bustard [66–68] and several ungulates [24,25], suggesting that a wide range of lekking species may be vulnerable to any disturbance of their natural display behaviour, especially those exposed to higher temperatures. These impacts are going to be compounded by the likely future restrictions in breeding range of these species in Iberia, and the fact that grassland species have limited ability within unsheltered habitats, to buffer elevated temperatures with behaviour thermoregulation [69].

This work has shown how global warming may affect important behavioural mechanisms using the mating system of a lekking grassland bird species as an example. Behavioural response to elevated temperatures may affect energetically-costly breeding behaviours in a range of mammals and birds [70], highlighting the need for further work to understand the underlying mechanisms of such climate-species dynamics as well as accurately gauge the implications these changes may have at a population level. The study has also provided a reproducible example on how accelerometer data paired with a rigorous method for classifying behaviours and an appropriate statistical analysis, can be used to answer specific research questions with important conservation inferences for various taxonomic groups.

## Supporting information

S1 Fig Relative effectiveness of classification of male little bustard display behaviour by the various AcceleRater models. The precision is the probability that an assigned behaviour is correctly classified, and the recall is the probability that a sample with a particular behaviour will be correctly classified.
(PNG)

S2 Fig Mean acceleration (ms^-2^) of 13 video classified display sequences for each of the seven seconds. Error bars represent standard deviation.
(TIFF)

S3 Fig GAMM showing the partial effect of Julian date on display activity (proportion of display events in each day) of 17 male little bustards tagged at five sites in Extremadura (Western Spain) and Alentejo (Eastern Portugal) in SW Iberia. The model incorporates average daytime temperature and Julian date as fixed effects and bird identity as random effect. Dashed black lines represent 95% confidence intervals.
(TIFF)

S1 Table Dependent and predictor variables included in Generalised Additive Mixed Models to explain male little bustard display behaviour. (DOCX)
