# Supplementary figures and images for: Feeling the heat: Elevated temperature affects male display activity of a lekking grassland bird
Source: PLoS One. 2019 Sep 16;14(9):e0221999. doi: 10.1371/journal.pone.0221999 (PMC6746384; doi:10.1371/journal.pone.0221999)

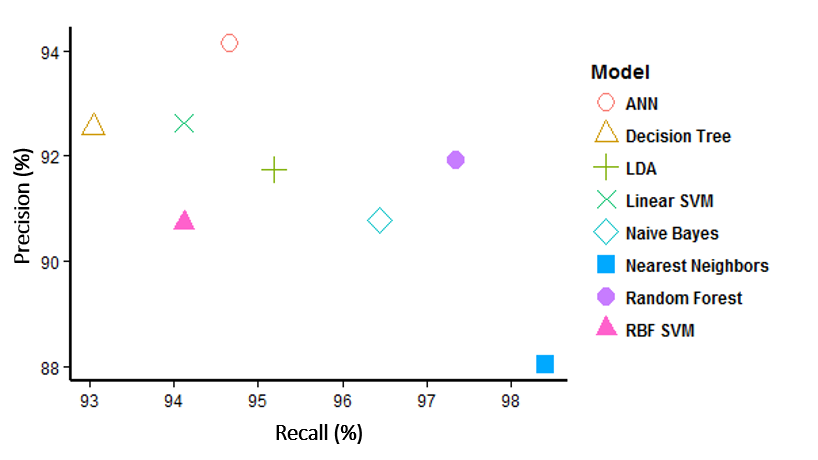

Supplement: S1 Fig — The precision is the probability that an assigned behaviour is correctly classified, and the recall is the probability that a sample with a particular behaviour will be correctly classified. (PNG) [file pone.0221999.s001.png]

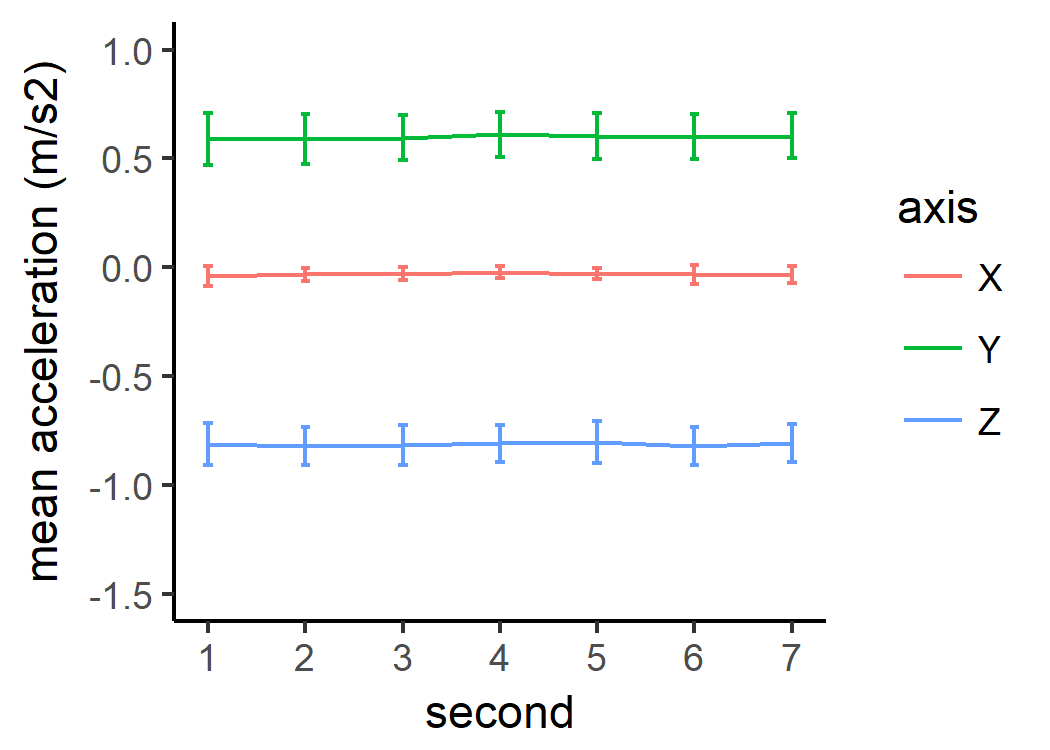

Supplement: S2 Fig — Error bars represent standard deviation. (TIFF) [file pone.0221999.s002.tiff]

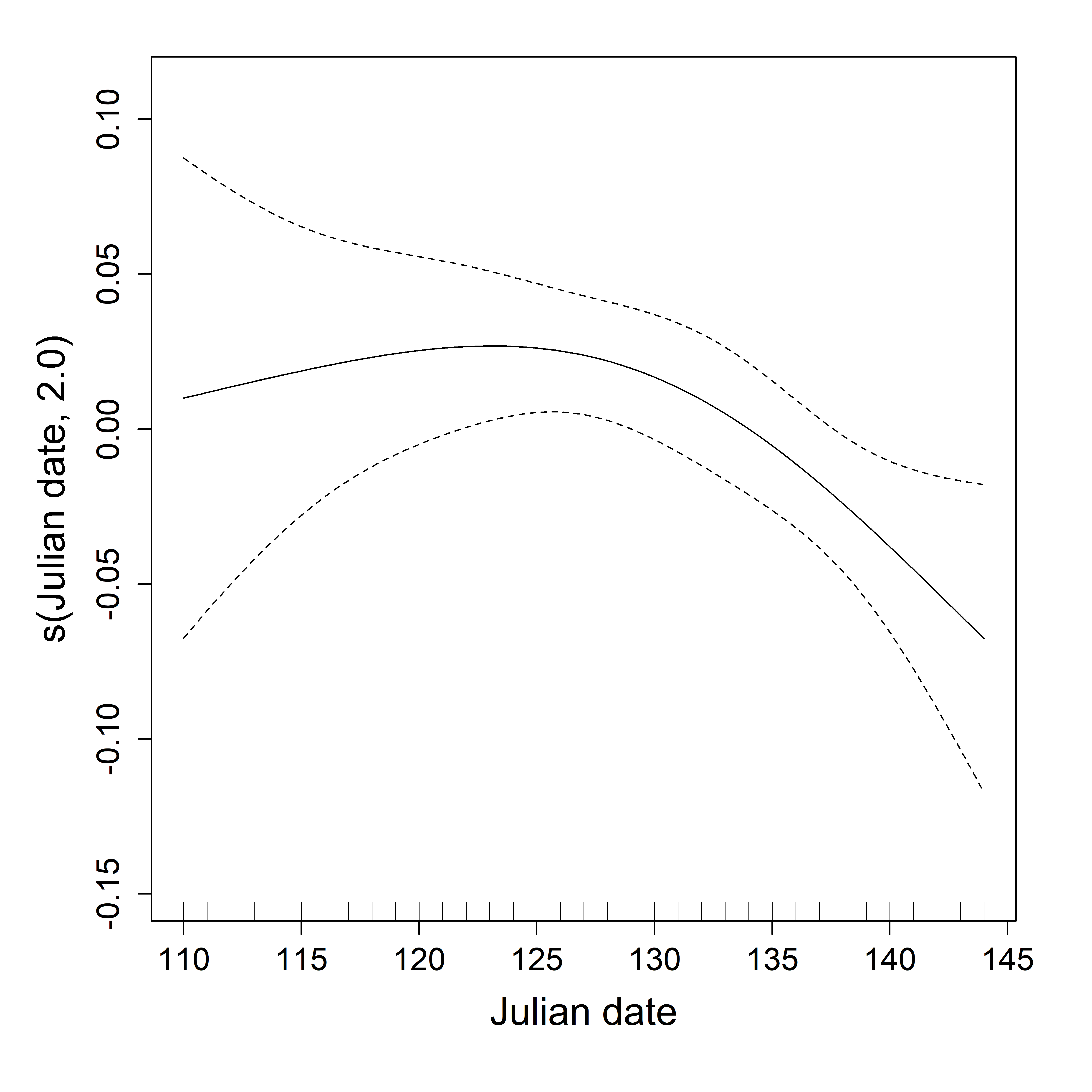

Supplement: S3 Fig — The model incorporates average daytime temperature and Julian date as fixed effects and bird identity as random effect. Dashed black lines represent 95% confidence intervals. (TIFF) [file pone.0221999.s003.tiff]
