# Supplementary material for: Feeling the heat: Elevated temperature affects male display activity of a lekking grassland bird
Source: PLoS One. 2019 Sep 16;14(9):e0221999. doi: 10.1371/journal.pone.0221999 (PMC6746384; doi:10.1371/journal.pone.0221999)
Supplement: S1 Table — (DOCX) [file pone.0221999.s004.docx]

| **Variable** | **Type** | **Description** |
| --- | --- | --- |
| Time | Continuous numerical variable | Hour of the day, from 5:00 – 21:00 hours; e.g. 6 represents time between 6:00 – 6:59 hours |
|  |  |  |
| Julian date | Discrete numerical variable | A measure of how far the display season has progressed. Julian date ranged from 107 (17th April) to 144 |
|  |  |  |
| Hourly temperature | Continuous | Average hourly ambient temperature (°C) at each site |
|  |  |  |
| Daily mean temperature | Continuous | Average daily ambient temperature (°C) for each bird between 5:00 – 21:00 hours |
|  |  |  |
| Bird identity | Categorical | Individual bird identification code |
|  |  |  |
|  |  |  |
| Display probability | Binary | Probability (0/1) of display behaviour for each recorded 10-second accelerometer sequence |
|  |  |  |
| Display activity | Proportion | Proportion of daily accelerometer sequences classified as display for each bird |

**S1 Table.** Dependent and predictor variables included in Generalised Additive Mixed Models to explain male little bustard display behaviour
